# Preparation and Characterization of Hemicellulose Films from Sugarcane Bagasse

**DOI:** 10.3390/ma13040941

**Published:** 2020-02-20

**Authors:** Roberta da Silva Braga, Matheus Poletto

**Affiliations:** 1 Chemical Engineering, University of Caxias do Sul (UCS), Caxias do Sul 95070-560, Brazil; rsbraga1@ucs.br; 2 Postgraduate Program in Engineering of Processes and Technologies (PGEPROTEC), University of Caxias do Sul (UCS), Caxias do Sul 95070-560, Brazil

**Keywords:** polymeric films, extraction, polysaccharide

## Abstract

Hemicellulose is one of the most common polysaccharides found in nature. Its use as a green and sustainable raw material for industries is desirable. In this work, an alkaline-alcoholic method was used to extract hemicelluloses from sugarcane bagasse. After extraction, films with 2%, 3% and 4% (w/v) hemicellulose were produced. The films’ morphology, thickness, water solubility, tensile properties and thermal stability were evaluated. The Fourier Transform Infrared Spectroscopy (FTIR) results reveal that the method used removes the hemicellulose from bagasse with a low concentration of lignin. The films presented a compact and dense structure with uniformity in thickness associated with higher solubility in water. The increase in hemicellulose content increased tensile strength, but reduced the tensile strain of the films. Thermogravimetric analysis indicated that the increase in hemicellulose content reduced the films’ thermal stability. Thus, these films may act as useful, biodegradable and environmentally friendly materials for engineering applications.

## 1. Introduction

Recently, consumer demand for healthier, safer and natural products has increased [1,2,3]. Consequently, in recent years, there has been growing interest in the development of materials from natural polymers, particularly those obtained from renewable resources [4,5]. Biopolymers isolated from plant-biomass, such as agricultural waste, are the usual alternative source of fillers for composites, biodegradable packaging and biomedical engineering materials, among others [1,6].

Sugar mills generate approximately 270 kg of bagasse (50% moisture) per metric ton of sugarcane [7]. Half of this waste is used to generate the energy required for the production of sugar and in fuel ethanol plants [7,8]. The other half is stockpiled, which constitutes an environmental problem due to the risk of spontaneous combustion [7] and vector proliferation. In Brazil alone, during the crop of 2018–2019, 621 million tons of sugarcane were processed in sugar mills [9], generating approximately 200 million tons of sugarcane bagasse. The bagasse provides a low-cost feedstock for production of fuels and chemicals, derived from a renewable resource. This provides a sustainability advantage from environmental, economic and social points of view [6,7,8].

Sugarcane bagasse is a rich source of polysaccharides. Cellulose and hemicellulose represent about 70% of the bagasse, while lignin and other minor compounds, such as extractives and minerals, constitute the remaining part [8]. Bagasse normally contains 30–35 wt.% hemicellulose: an amorphous polymer composed of xylose, galactose, mannose, arabinose, other sugars and their uronic acids [7]. The extraction of hemicelluloses from sugarcane bagasse is an interesting alternative way to convert this waste into value-added products for chemical, agricultural, medical and pharmaceutical applications [8]. The applications of hemicellulose-derived materials include packaging, important chemicals such as xylan, and other saccharides [8]. However, hemicelluloses are intrinsically bound to cellulose and lignin in the plant cell wall, so it is difficult to separate them without causing a modification in their structure [8,10].

Several treatments have been used for hemicellulose extraction. Sun et al. used alkaline peroxide treatment to remove hemicelluloses from sugarcane bagasse and observed the formation of a backbone of xylose branched through arabinofuranosyl and grucopyranosyl units [11]. Xu et al. also obtained hemicelluloses from sugarcane bagasse, but the authors compared alkali (NaOH) and acid organic (1,4-dioxane) treatment after removal of hemicelluloses from bagasse using toluene-ethanol mix as a solvent [7]. Alkaline treatment generated hemicelluloses with higher molecular weights and a more linear structure than acid treatment, since hemicelluloses degraded after acid organic treatment [7]. Brienzo et al. evaluated the extraction of hemicellulose using alkaline peroxide prior to washing the bagasse with ethylenediamine tetraacetic acid [8]. They obtained hemicelluloses in the absence of magnesium sulfate, which can promote hemicellulose degradation [8].

Other lignocellulosic materials have also been used to obtain hemicelluloses. Cerqueira et al. obtained galactomannans from the seeds of four different species of Leguminosae *(Adenanthera pavonina*, *Caesalpinia pulcherrima*, *Gleditsia triacanthos* and *Sophora japonica*) using aqueous extraction followed by precipitation and purification with ethanol [4]. Börjesson et al. obtained arabinoxylan from wheat bran, which contains approximately 18 wt.% hemicelluloses, using alkaline extraction. However, given the high quantities of bagasse generated by sugar mills and the high quantities of hemicellulose in this waste (30–35 wt.%) an interesting possibility is to create valued-added materials from sugarcane bagasse.

Thus, the main objective of this work was to develop and characterize the properties of hemicellulose films produced from sugarcane bagasse using an alkali-alcoholic extraction. The morphological, thermal, chemical and mechanical properties of the films were evaluated.

## 2. Methodology

### 2.1. Materials

Sugarcane bagasse was obtained from a local distillery (Bento Gonçalves, Brazil). It was first dried in an oven with air circulation for 4 h at 90 °C and then ground in a knife mill to pass a 0.7 mm size screen. Afterwards, it was de-waxed with ethanol in a Soxhlet apparatus for 6 h. All chemicals used (ethanol, NaOH and HCl) were analytical grade, purchased from Vetec Chemistry (Rio de Janeiro, Brazil).

### 2.2. Alkali Extraction

Wax-free bagasse (100 g) was extracted with 2.5 L of 0.5 M NaOH over 2 h at 23 °C with magnetic stirring. After that, the bagasse was separated from the supernatant by filtration. The supernatant solution was neutralized to pH 5.5 with dropwise addition of 6 M HCl in an ice bath. The solution was kept at 4 °C for 24 h. The solution was centrifuged at 4000 rpm for 15 min and then 3 volumes of ethanol were added to the still solubilized hemicelluloses. After precipitation, the sample was centrifuged again. The hemicelluloses precipitated were dried at 23 °C for 24 h to form pellets.

### 2.3. Hemicelluloses Characterization

FTIR spectra of the hemicelluloses were obtained from a Nicolet IS10 Thermo Scientific spectrometer (Waltham, MA, USA) using a KBr disc containing 1% finely ground sample. The spectra were recorded using 32 scans at resolutions of 4 cm^−1^ from 4000–400 cm^−1^.

### 2.4. Films Preparation

The hemicelluloses were dissolved in distilled water to form solutions with concentrations of 2%, 3% and 4% (w/v). The solutions were then stirred at 600 rpm at 23 °C for 6 h with a magnetic stirrer. Afterwards, the film solutions were cast and dried at 23 °C for 72 h. In the next step, the films were placed in a desiccator for 24 h before the tests.

### 2.5. Films Characterization

The morphology of the films was investigated using scanning electron microscopy in a FEG SEM Tescan Mira 3 (Brno, Czech) at 10 kV. The samples were sputter coated with gold before examination. The film thickness was measured using a digital thickness gauge (Mitutoyo, Japan). The measurements were taken at 10 different points on the films. The solubility test was done in triplicate on samples measuring 2 cm^2^. The samples were pre-dried in an oven for 24 h at 50 °C. Subsequently, the samples were weighed to obtain the initial film dry weight (m_i_). The samples were then immersed in 50 mL of distilled water at room temperature and stirred at 60 rpm for 2 h. The remaining pieces of the samples were filtered and dried at 70 °C for 24 h and weighed again to obtain the final weight (m_f_). The water solubility (%) was calculated as presented in Equation (1):

Solubility (%) = [(m_i_ – m_f_)/m_i_] × 100 (1)

The mechanical properties (tensile strength and elongation at break) were measured using an EMIC DL 2000 machine (São José dos Pinhais, Brazil) according to ASTM D882. The crosshead speed used was 5 mm·min^−1^. Five specimens measuring 40 mm × 5.3 mm were tested for each formulation evaluated. The thermogravimetric analyses of the films were carried out using a TGA-50 (Shimadzu, Japan). Nearly 5 mg of sample was used for each experiment with air gas at a flow rate of 63 mL·min^−1^. The temperature range and heating rate were 20–600 °C and 10 °C·min^−1^, respectively.

## 3. Results and Discussion

### 3.1. Alkali Extracted Hemicellulose Characterization

**Figure 1:**
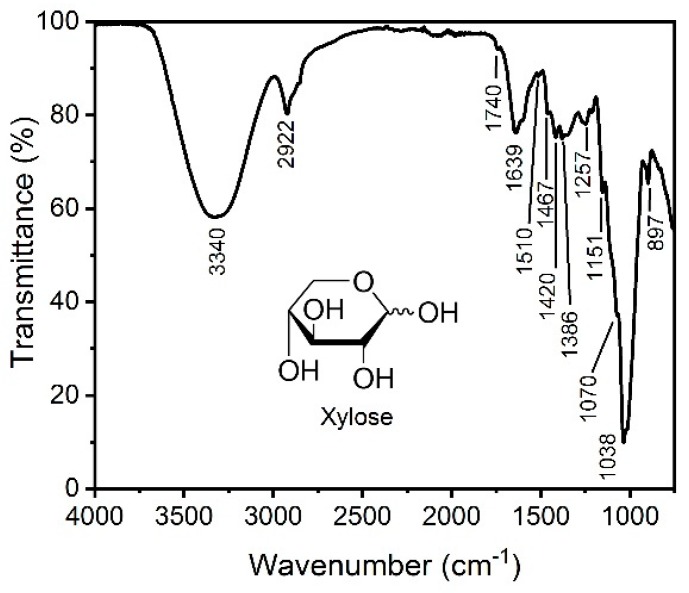
FTIR spectrum of extracted hemicelluloses from sugarcane bagasse.

The FTIR spectrum of hemicellulose extracted from sugarcane bagasse is shown in Figure 1. The broad band at 3340 cm^−1^ is attributed to hydroxyl groups present in the sugars that composed the hemicelluloses, as exemplified by the OH groups in the xylose molecule in Figure 1, and also to the water involved in hydrogen bonding [11]. The band detected at 2922 cm^−1^ is a result of C–H stretching vibrations due to –CH_2_ groups [11]. In addition, the bands at 1386 cm^−1^ and 1257 cm^−1^ are a result of C–H bending [11]. The strong signal at 1639 cm^−1^ is attributed to absorbed water [11,12]. A weak signal at 1740 cm^−1^ implies that the extracted hemicelluloses contain small amounts of acetyl, uronic, and ester groups, or may contain ester bonds of the carboxylic groups presented in ferulic and/or p-coumaric acids [11,12]. The band at 1510 cm^−1^ is attributed to aromatic skeletal vibrations associated with lignin [11,12], which indicate that extracted hemicelluloses were contaminated with small amounts of bound lignin. Bands related to –CH_2_ stretching vibrations were observed at 1467 and 1420 cm^−1^ [12]. The prominent band at 1038 cm^−1^ is assigned to C–O and C–C stretching and to the glycosidic linkage contributions [12,13]. The band at around 1040 cm^−1^ is a clear indication of hemicelluloses originating from xylans [13]. The band at 897 cm^−1^ is generally associated with β-glycosidic bonds between sugars [13].

These FTIR results indicate that the solution of 0.5 M NaOH under the conditions adopted in this work extracted hemicelluloses, mainly xylan, with a small amount of lignin. The treatment used promoted the scission of ether bonds between lignin and hemicellulose from the cell walls of sugarcane bagasse, causing an elevated dissolution of the polymeric structures composing the hemicelluloses from the aqueous media [7]. The extraction of hemicelluloses normally involves the hydrolysis of ester linkages present in polysaccharides under alkaline conditions to liberate the hemicelluloses from the lignocellulosic matrix [11]. However, the liberation of the hemicellulose sugars from the plant cell walls is affected by the presence of lignin networks as well as ester and ether lignin–hemicellulose linkages [11]. On the other hand, the hydroxyl ions also cause swelling of cellulose and disruption of intermolecular hydrogen bonds between cellulose and hemicelluloses, which results in its solubilization to the aqueous media [7]. During the extraction, not only lignin, but also cellulose can be removed from the lignocellulosic materials, because of the random scission caused by the hydroxyl ions on the structure of the plant cell wall. In addition, the alkaline treatment may cause branching on the structure of hemicelluloses [7].

### 3.2. Microstrucutre of Hemicellulose Fims

**Figure 2:**
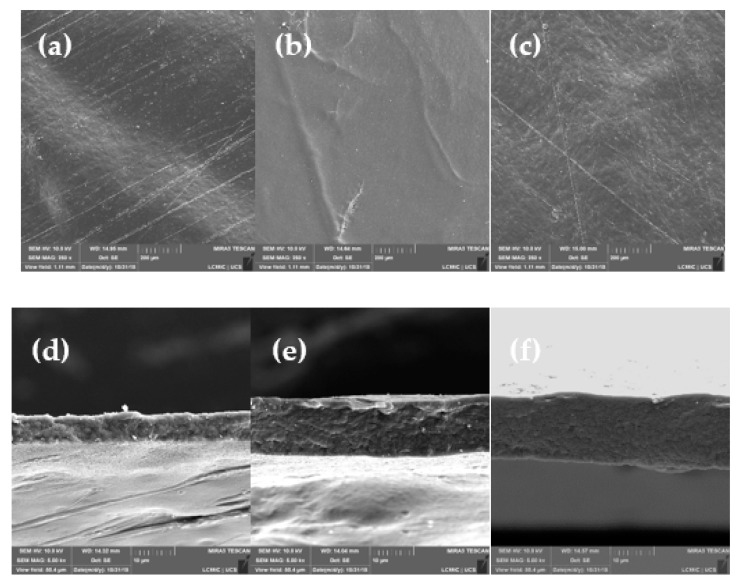
SEM images of the surface (250×) and cross-section (5000×) hemicellulose films: (**a**,**d**) 2 %(w/v), (**b**,**e**) 3 % (w/v), (**c**,**f**) 4 % (w/v).

The surface and cross-section SEM images of the hemicellulose films developed are shown in Figure 2.

The films presented a compact and dense structure with a reduced surface roughness. The cross-section images reveal a structure formed of several layers with a thin membrane near to the film surface. The hydroxyl groups presented in the hemicelluloses can promote a rigid hydrogen-bonded network resulting in a compact and dense film. Huang et al. also observed the same dense and reduced surface porosity in hemicellulose-based films reinforced with nanocrystalline cellulose [14]. The authors attribute the dense structure of the films to the hydrogen bonds formed between hemicelluloses [14]. Prajapati et al. also reported that hydrogen bond formation is one of the more important characteristics of hemicelluloses [15].

### 3.3. Thickness and Solubility of Hemicellulose Films

Thickness is an important parameter considering the future use of these materials as packaging, since mechanical properties and gas barrier properties are controlled by film thickness [6]. Table 1 presents the thickness and solubility of the hemicellulose films. The thickness of the films ranged from 0.035 mm to 0.040 mm, showing the uniformity of the films produced in this work. Thickness control is necessary to ensure the uniformity, precision and accuracy of the film-making process [5]. The increase in hemicellulose concentration did not cause an increase in film thickness, which may indicate that the drying process was uniform for all samples in removing entrapped water. Entrapped water can cause thickness swelling and affect the film thickness.

Solubility in aqueous solutions is another relevant property of packaging films [6]. The films must present low water solubility to enhance product integrity [6]. However, in some cases (such as food and drug coating), a high film solubility in water may be beneficial [6]. The films showed a decrease in solubility when hemicellulose content increased. This behavior might be explained by the increased lignin content present in the films with increased hemicellulose. Lignin is a hydrophobic material and may reduce water absorption [16,17]. However, the solubility of the films is much higher than other polymeric materials generally used for package applications. This can be lowered with the addition of hydrophobic compounds, such as lignin.

### 3.4. Mechanical Properties

**Figure 3:**
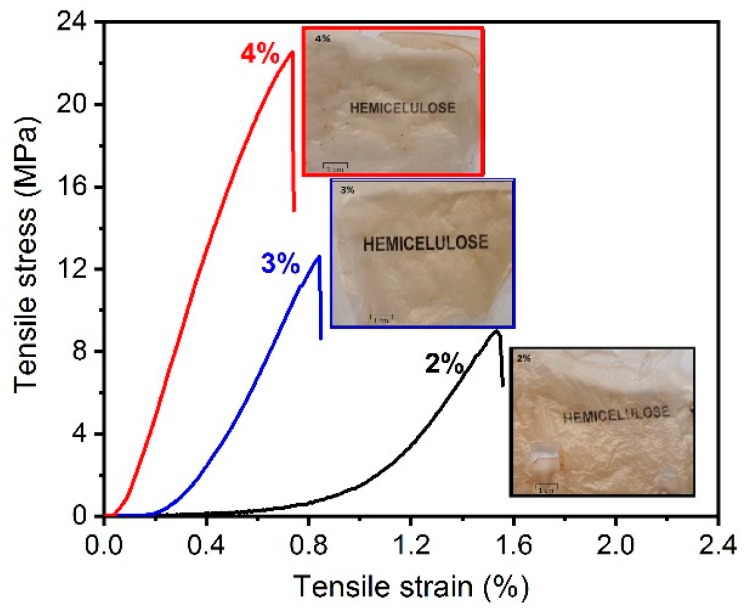
Tensile strain curves of hemicellulose films.

The typical stress–strain curves of hemicellulose films are shown in Figure 3. Increased hemicellulose content resulted in films becoming more rigid but brittle. The obtained films were transparent with a yellowish-brown appearance, as can be seen in Figure 3. 

The tensile stress, tensile strain at break and elastic moduli of films are presented in Table 2. The increase in hemicellulose concentration results in films with higher tensile strength and elastic modulus. For biomacromolecules, the mechanical strength mainly depends on the hydrogen bonds [10]. Therefore, when hemicellulose content increases there are more hydroxyl groups that may form hydrogen bonds, which results in films with higher mechanical strength. In addition, when hemicellulose content increases, the film grammage also increases. This might result in better stress transfer to the hemicellulose chains, improving the mechanical performance of the films. Mechanical properties can also vary due to the type and composition of the hemicelluloses [18] obtained during the extraction process.

The tensile strain at break was reduced with increased hemicellulose content. Bioplastics, including polysaccharides, typically presented very brittle behavior with tensile strains at break lower than 10% [3], probably due to the rigid hydrogen bonding network formed between inter- and intramolecular hemicellulose chains.

### 3.5. Thermogravimetric Analysis

**Figure 4:**
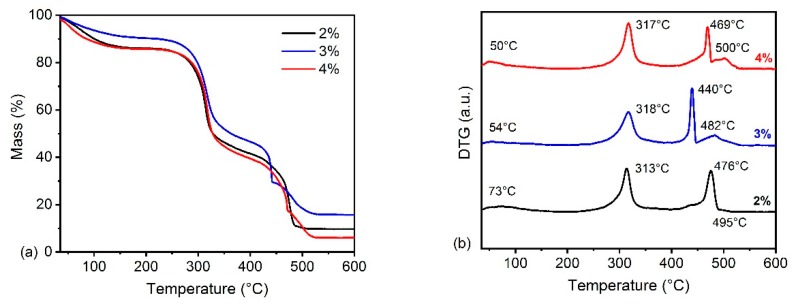
Thermogravimetric curves of hemicellulose films.

The thermogravimetric (TG) and first derivative thermogravimetric (DTG) curves of the films are shown in Figure 4. The samples underwent four weight loss events. The first was related to the loss of absorbed and structural water [19,20], which is in turn associated with the hydrophilic nature of the functional groups of each polysaccharide [1]. This occurred between 50 and 100 °C. The second event was the main weight loss of hemicellulose and occurred between 220 and 340 °C [21,22] with a main degradation peak at around 320 °C. Hemicellulose is formed of various saccharides with a random amorphous structure rich of branches, which are easily decomposed in an air atmosphere [22,23]. These saccharides are decomposed to volatiles including CO, CO_2_ and some hydrocarbons with low molecular mass, such as CH_4_, C_2_H_4_ and C_2_H_6_ [23]. The other peaks, at 440–500 °C, may be associated with the decomposition of hydrocarbons formed during hemicellulose degradation and with the lignin fraction presented in the films’ composition, as also discussed in the FTIR results.

In general, increases in hemicellulose content promoted a reduction in the thermal stability of the films. This fact may be associated with increased oxygen content in the films. This can provide sites for initiating the degradation process at lower temperatures, since the degradation of low molecular compounds, present in hemicelluloses (xylose, mannose, glucose, etc.), may accelerate the main hemicellulose degradation [17,19]. The integral decomposition temperature (IPDT) proposed by Doyle [24] correlates with the volatile parts of polymeric materials [25,26], and was used in this work to estimate the inherent thermal stability of hemicellulose films. IPDT was calculated from:

IPDT (°C) = A^*^ K^*^ (T_f_ – T_i_) + T_i_ (2)

A^*^ = (S_1_ + S_2_)/(S_1_ + S_2_ + S_3_) (3)

**Figure 5:**
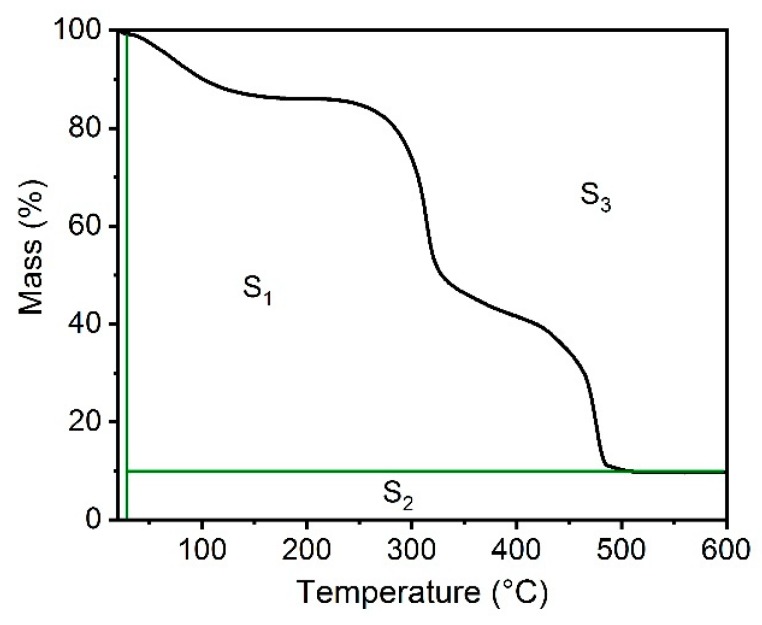
Schematic representation of S_1_, S_2_ and S_3_.

K^*^ = (S_1_ + S_2_)/S_1_ (4)

where T_i_ and T_f_ are the initial and final experimental temperatures. Figure 5 shows a representation of S_1_, S_2_ and S_3_ areas used to calculate A^*^ and K^*^.

The IPDT values are presented in Table 3. In theory, higher thermal stability leads to higher IPDT values [27]. The film with 3% hemicellulose showed the highest value, followed by films with 2% and 4%. This behavior is in agreement with thermogravimetric results, and probably indicates that more volatile compounds were liberated during the degradation of the film with 4% hemicellulose, reducing its thermal stability. In addition, a non-linear relationship between thermal stability and concentration was observed. The balance between oxygen content and hydrogen bond formation is probably responsible for this result. When the hemicellulose content is the lowest, the hydrogen bond formation is low, which may result in low thermal stability. On the other hand, when the hemicellulose content is highest, hydrogen bond formation increases, but the oxygen content also increases. This probably results in low thermal stability, since several degradation sites are available. Thus, the film with 3% hemicellulose presented the highest thermal stability. The method proposed by Broido [28] was used to obtain the activation energy (Ea) of the films during thermal degradation. The model used is an uncomplicated and relatively accurate method [28,29] for calculating Ea according to Equation (5):

ln [−ln(1 − α)] = −Ea/(RT) + const.(5)

where *α* is the conversion corresponding to a particular stage of decomposition (in this study, only considered during hemicellulose decomposition), *T* is the absolute temperature in Kelvin and *R* is the universal gas constant (8.31 J mol^−1^ K^−1^). In this method, plotting ln [−ln(1 − α)] versus (1000/T) produces straight lines used to determine Ea from their slopes (−Ea/R). The Ea values and their correlation coefficients are listed in Table 3.

The film with 3% hemicellulose presented the highest Ea value, in agreement with the highest IPDT value and highest thermal stability observed in thermogravimetric analysis. The sample with 4% hemicellulose showed a higher Ea value than the film with 2% hemicellulose. Possibly, more energy must be used to break the hydrogen bonds formed in the film with 4% hemicellulose. However, as explained above, the degradation of the film with 4% hemicellulose may liberate more volatile compounds which results in low thermal stability, also reducing the IPDT values. The Ea values obtained in this work are in conformity with the values presented by Cao et al. for xylan [30]. The authors obtained an Ea equal to 73.85 kJ mol^−1^ and a R^2^ value of 0.939 [30].

## 4. Conclusions

The thickness and solubility of the films developed are promising properties for their use as packaging or food and drug coating. As the hemicellulose content of the films increased, tensile strength and elastic modulus also increased, mainly due to an increase in the hydrogen bonding networks formed between hemicelluloses. However, thermal stability was reduced with increased hemicellulose content. The results obtained in this work suggest that the films have promising properties for use in food-related and packaging applications. Hemicellulose is an available, edible, sustainable, biodegradable, low-cost, non-toxic and environmentally friendly material that has several potential uses in industrial applications.

## Figures and Tables

**Table 1:** Thickness and solubility of the hemicellulose films.

Samples (w/v)	Thickness (mm)	Solubility (%)
2%	0.040 ± 0.006	92.7 ± 3.5
3%	0.036 ± 0.005	91.3 ± 2.2
4%	0.035 ± 0.011	85.6 ± 8.1

**Table 2:** Tensile results of the hemicellulose films developed.

Samples (w/v)	Tensile Strength (MPa)	Tensile Strain at Break (%)	Elastic Modulus (MPa)
2%	9.2 ± 1.9	1.3 ± 0.4	8.9 ± 1.1
3%	12.4 ± 0.9	0.8 ± 0.1	23.5 ± 1.7
4%	22.3 ± 3.3	0.7 ± 0.2	38.9 ± 2.2

**Table 3:** Integral decomposition temperature (IPDT) values and activation energy of the films using the Broido method.

Samples (w/v)	IPDT (°C)	Ea (kJ mol^−1^)	R^2^ Values
2%	715	67.2	0.950
3%	794	84.8	0.992
4%	663	73.1	0.951

## References

[1] Cerqueira M.A., Souza B.W.S., Simões J., Teixeira J.A., Domingues M.R.M., Coimbra M.A., Vicente A.A. (2011). Structural and thermal characterization of galactomannans from non-conventional sources. Carbohydr. Polym..

[2] Mohomane S.M., Motaung T.E., Revaprasadu N. (2017). Thermal and degradation kinetics of sugarcane bagasse and soft wood cellulose. Materials.

[3] Börjesson M., Westman G., Larsson A., Ström A. (2019). Thermoplastic and flexible films from arabinoxylan. ACS Appl. Polym. Mater..

[4] Cerqueira M.A., Pinheiro A.C., Souza B.W.S., Lima A.M.P., Miranda C.R.C., Teixeira J.A., Moreira R.A., Coimbra M.A., Gonçalves M.P., Vicente A.A. (2009). Extraction, purification and characterization of galactomannans from non-traditional sources. Carbohydr. Polym..

[5] Mendes F.R.S., Bastos M.S.R., Mendes L.G., Silva A.R.A., Sousa F.D., Monteiro-Moreira A.C.O., Cheng H.N., Biswas A., Moreira R.A. (2017). Preparation and evaluation of hemicellulose films and their blends. Food Hydrocoll..

[6] Goksu E.I., Karamanlioglu M., Bakir U., Yilmaz L., Yilmazer U. (2007). Production and characterization of films from cotton stalk xylan. J. Agric. Food Chem..

[7] Xu F., Sun J.X., Liu C.F., Sun R.C. (2006). Comparative study of alkali- and acidic organic solvent-soluble hemicellulosic polysaccharides from sugarcane bagasse. Carbohydr. Res..

[8] Brienzo M., Siqueira A.F., Milagres A.M.F. (2009). Search for optimum conditions of sugarcane bagasse hemicellulose extraction. Biochem. Eng. J..

[9] Unica. http://www.unicadata.com.br/historico-de-producao-e-moagem.php?idMn=31&tipoHistorico=2&acao=visualizar&idTabela=2333&produto=cana&safraIni=2018%2F2019&safraFim=2018%2F2019&estado=RS%2CSC%2CPR%2CSP%2CRJ%2CMG%2CES%2CMS%2CMT%2CGO%2CDF%2CBA%2CSE%2CAL%2CPE%2CPB%2CRN%2CCE%2CPI%2CMA%2CTO%2CPA%2CAP%2CRO%2CAM%2CAC%2CRR.

[10] Zhong L.-X., Peng X.-W., Yang D., Cao X.-F., Sun R.-C. (2013). Long-chain anhydride modification: A new strategy for preparing xylan films. J. Agric. Food Chem..

[11] Sun J.X., Sun X.F., Sun R.C., Su Y.Q. (2004). Fractional extraction and structural characterization of sugarcane bagasse hemicelluloses. Carbohydr. Polym..

[12] Ruiz H.A., Cerqueira M.A., Silva H.D., Rodríguez-Jasso R.M., Vicente A.A., Teixeira J.A. (2013). Biorefinery valorization of autohydrolysis wheat straw hemicellulose to be applied in a polymer-blend film. Carbohydr. Polym..

[13] Egüés I., Eceiza A., Labidi J. (2013). Effect of different hemicelluloses characteristics on film forming properties. Ind. Crops Prod..

[14] Huang B.H., Tang Y., Pei Q., Zhang K., Liu D., Zhang X. (2018). Hemicellulose-based films reinforced with unmodified and cationically modified nanocrystalline cellulose. J. Polym. Environ..

[15] Prajapati V.D., Jani G.K., Moradiya N.G., Randeria N.P., Nagar B.J., Naikwadi N.N., Variya B.C. (2013). Glactomannan: A versatile biodegradable seed polysaccharide. Int. J. Biol. Macromol..

[16] Demosthenes L.C.C., Nascimento L.F.C., Monteiro S.N., Costa U.O., Garcia Filho F.C., Luz F.S., Oliveira M.S., Ramos F.J.H.T., Pereira A.C., Braga F.O. (2019). Thermal and structural characterization of buriti fibers and their relevance in fabric reinforced composites. J. Mater. Res. Technol..

[17] Poletto M., Zattera A.J., Santana R.M.C. (2012). Structural differences between wood species: Evidence from chemical composition, FTIR spectroscopy, and thermogravimetric analysis. J. Appl. Polym. Sci..

[18] Sabiha-Hanim S., Siti-Norsafurah M. (2012). Physical properties of hemicellulose films from sugarcane bagasse. Procedia Eng..

[19] Poletto M., Ornaghi Júnior H.L., Zattera A.J. (2014). Native cellulose: Structure, characterization and thermal properties. Materials.

[20] Ornaghi Júnior H.L., Moraes A., Poletto M., Zattera A.J., Amico S. (2016). Chemical composition, tensile properties and structural characterization of buriti fiber. Cellulose Chem. Technol..

[21] Telmo C., Lousada J. (2011). The explained variation by lignin and extractive contents on higher heating value of wood. Biomass Bioenergy.

[22] Yang H., Yan R., Chen H., Zheng C., Lee D.H., Liang D.T. (2006). In-depth investigation of biomass pyrolysis based on three major components: Hemicellulose, cellulose and lignin. Energy Fuels.

[23] Yang H., Yan R., Chen H., Lee D.H., Zheng C. (2007). Characterization of hemicellulose, cellulose and lignin pyrolysis. Fuel.

[24] Doyle C.D. (1961). Estimating thermal stability of experimental polymers by empirical thermogravimetric analysis. Anal. Chem..

[25] Chiang C.-L., Chang R.C., Chiu Y.C. (2007). Thermal stability and degradation kinetics of novel organic/inorganic epoxy hybrid containing nitrogen/silicon/phosphorus by sol–gel method. Thermochim. Acta.

[26] Park S.-J., Cho M.-S. (2000). Thermal stability of carbon-MoSi_2_-carbon composites by thermogravimetric analysis. J. Mater. Sci..

[27] Ornaghi Júnior H.L., Poletto M., Zattera A.J., Amico S. (2014). Correlation of the thermal stability and the decomposition kinetics of six different vegetal fibers. Cellulose.

[28] Broido A. (1969). A simple, sensitive graphical method of treating thermogravimetric analysis data. J. Polym. Sci. A.

[29] Yassin A.Y., Mohamed A.-R., Abdelrazek E.M., Morsi M.A., Abdelghany A.M. (2018). Structural investigation and enhancement of optical, electrical and thermal properties of poly(vinyl chloride-co-vinyl acetate-co-2-hydroxypropyl acrylate)/graphene oxide nanocomposite. J. Mater. Res. Technol..

[30] Cao W., Li J., Martí-Rosselló T., Zhang X. (2019). Experimental study on the ignition characteristics of cellulose, hemicellulose, lignin and their mixtures. J. Energy Inst..

